# Supplementary material for: Neural processing of rhythmic speech by children with developmental language disorder (DLD): An EEG study
Source: Imaging Neurosci (Camb). 2024 Dec 5;2:imag-2-00382. doi: 10.1162/imag_a_00382 (PMC12315765; doi:10.1162/imag_a_00382)
Supplement: Supplementary Material [file imag_a_00382-supp.pdf]

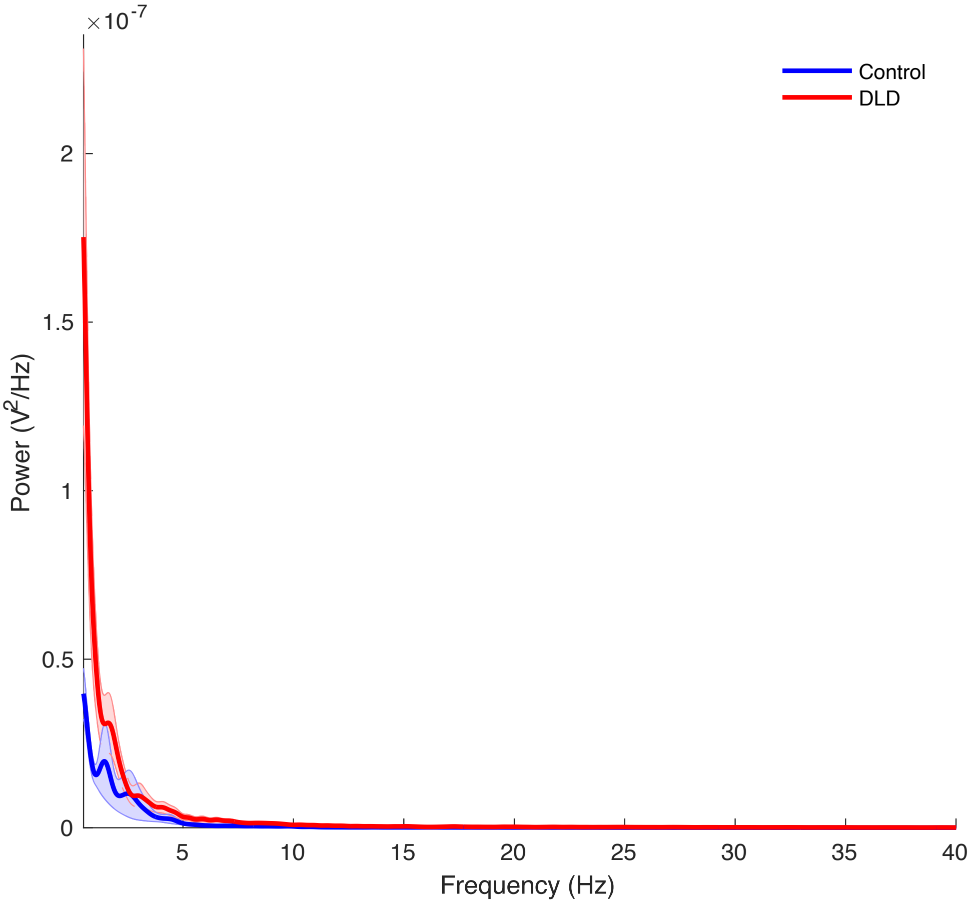

Figure S1. Broad-band spectral power in the entrainment period. The shaded areas denote the standard error of the mean for each group.
